# An explorative cross-sectional analysis of mental health shame and help-seeking intentions in different lifestyles

**DOI:** 10.1038/s41598-023-37955-8

**Published:** 2023-07-04

**Authors:** Claudia Helmert, Toni Fleischer, Sven Speerforck, Christine Ulke, Laura Altweck, Stefanie Hahm, Holger Muehlan, Silke Schmidt, Hans J. Grabe, Henry Völzke, Georg Schomerus

**Affiliations:** 1 Department of Psychiatry and Psychotherapy, University of Leipzig, Medical Center, Leipzig, Germany; 2 Department of Health and Prevention, Greifswald University, Greifswald, Germany; 3 Department of Psychiatry and Psychotherapy, Greifswald University, Medical Center, Greifswald, Germany; 4 Institute for Community Medicine, Greifswald University, Medical Center, Greifswald, Germany

**Keywords:** Health care, Health policy, Health services, Public health, Psychology and behaviour, Human behaviour

## Abstract

To identify und support particular target groups for mental health prevention, we explore the links between shame and help-seeking intentions concerning mental health in different lifestyles (based on socioeconomic status as well as health-related behaviors). Lifestyles were operationalized by nine confirmatory, homogenous clusters of the sample. These clusters are based on individuals’ similarities in sociodemographic aspects and health behavior. Analyses included *t* tests, Chi-square, ANOVA, regressions investigating in sociodemographic characteristics. Hierarchical linear models examining cross-sectional associations of shame and willingness to seek help for different lifestyles of participants of the Study of Health in Pomerania (SHIP-START-1 and SHIP-START-3, data collected 2002–2006 and 2014–2016; n = 1630). Hierarchical linear models showed small context effects for lifestyle-related associations of shame and willingness to seek help. For younger as well as male participants, lifestyles indicated different associations of shame and help-seeking intentions: Especially the lifestyles with unhealthy behaviors and high as well as low socioeconomic status resulted in higher shame being associated with low help-seeking intentions in case of mental illness. Lifestyle clusters might be a useful tool to identify marginalized groups with unhealthy behaviors, which should be addressed by interventions and prevention programs.

## Introduction

There are remarkably high proportions of persons with mental illness^1,2^ not seeking help^3,4^. Though, the intentions to seek help as coping with diseases are manifold, like communicating with other people, to get support not only by experts but also family and friends^5^. Several studies found barriers hindering people’s help seeking like role expectations of being male^3,6^ and higher age^7,8^. Not only sociodemographic aspects but also low mental health literacy^9,10^ and shame^11^ are examined as relevant barriers as well. Taking into consideration that socioeconomic position is linked to health-related behavior and status^12^ as well as to mortality^13,14^, the present paper aims to implement a health-related concept of different lifestyles to investigate in the association of shame and intended help-seeking^12^.

As mentioned research revealed, not only sociodemographic aspects play important roles in help-seeking research. To identify marginalized groups, to build a framework for analyzing the complexity and interactions of factors hindering people to seek help, social structures were considered^15^: Lifestyles help to analyze clusters for social structures based on similar behavioral routines and shaped identities, besides for instance milieus^16^. In general, multidimensional facets of living, working and interactions were considered for conceptualizing lifestyles^17^. The present paper investigates lifestyles by combining health-related behaviors and sociodemographic aspects to get information on individuals’ attitudes and habits^18^ concerning mental health.

One of these attitudes of interest refer to stigmatization. In detail, perceived discrimination^19^ and shame^20^ hinder help seeking, as well. Focusing on shame as the emotional aspect of self-stigma^21^ appeared important, because shame is the moral component influencing behavior and is socially embedded^22,23^. Shame refer to the ‘universal, adaptive and common emotional response to exposure of easily-hurt aspects of the self’^24^, while self-stigma equals internalized negative public attitudes^25^. Furthermore, studies identified the link between shame and mental health as well as associations with gender, income, and education^26,27^.

To find out about groups with particularly high levels of shame that hinder the willingness to seek help, the present study focuses on health-related lifestyles. As lifestyles were defined as combined behavioral routines^16,17^ we synthesize the known association between sociodemographic factors and health-related behaviors, to build a framework for detailed analyses on shame and willingness to seek help in case of mental illness. Further, these health-related lifestyle groups can be addressed by customized prevention campaigns to improve access to health care^28^.

### Hypothesis

Lifestyles as everyday practices and attitudes grouping people^16^ were build up on known association of sociodemographic aspects and health-related behaviors^12^. For lifestyles were associated with health status^29,30^ and were a potential tool for psychiatric research^15^, current hypothesis is: The associations of shame and willingness to seek professional help in case of mental illness vary by lifestyle characteristics (measured by health behavior and sociodemographic information).

## Methods

### Sample

Based on the Study of Health in Pomerania (SHIP) the sample referred to the third follow-up data collection (SHIP-START-3) from 2014 to 2016 (n = 1718) and was supplemented with SHIP-START-1 based on collected data from 2002 to 2006 (n = 3300)^31^. The population-based study design included interview assessments and comprehensive investigations in participants’ health status (like sleep monitoring)^31^. Inclusion criteria were of legal age (18 years) as well as residence in the study area Pomerania in northeast Germany and German nationality^31,32^. Details about the sample and response rate were described elsewhere^31,33^.

The final sample consisted of 1630 participants. Due to longitudinal SHIP-START data collection used herein, there were dropouts (n = 1758). Because collected information varied between different examination waves, current analyses based on a combination of information of two different time points (whereas educational and professional level from the first data collection from 2002 to 2006, where added to the information to the third follow-up as main data set with information about income, health-related behavior, stigma, shame and help-seeking intentions) without paying attention to temporality. Only participants who took part in both surveys were included in cross-sectional analyses. Regarding the final data set, cases with missing values in variables of interest were less than 5% (for level 1 and level 2 variables in multilevel analyses) and therefore excluded from calculations^34^.

### Operationalization of lifestyles, shame, and help-seeking intentions

Lifestyles were operationalized by confirmatory, homogenous cluster analyses^35^. In general, the lifestyles of interest referred to associations of tangible assets and health-related behavior^12,36^. Operationalization was based on the already known link of socioeconomic status (SES) and health-related behavior^14,37^. The sample was grouped by similarities in educational and professional level, income, smoking status as well as alcohol drinking behavior and physical activity leading to nine different lifestyles. These included all possible combinations of people’s characteristics of high, middle to low socioeconomic status and healthy, moderately healthy to unhealthy behaviors. The household income, educational level and professional status operationalized the SES Index^38^. Health behavior consisted of summarized dichotomously results for the following information: For participants’ daily smoking status (more than zero cigarettes equals unhealthy behavior^39^), alcohol consumption within the last month (number of days on which the participant drank alcohol, and how much, referring to reference values by guideline for risky drinking behavior stratified by sex^40^). Reported physical activity was part of lifestyle operationalization, as well. In the final step, each information on smoking, alcohol consumption was summarized by a binary variable (0 unhealthy, 1 healthy), and physical activity by three categories ranging from 0 unhealthy, 0.5 moderately healthy to 1 healthy. Sum scores of SES as well as health behavior led to population based quintiles which were pooled in three categories (low/middle/high) to have sufficient sample sizes^41,42^ for every lifestyle cluster, provided in the supplementary material (Table S1). To identify the association of intended help-seeking (“Would you seek professional help if you feel depressed for a long time or if you had other mental problems?”, 1 “not at all” to 5 “very much”) and shame (“Would you feel ashamed if you were mentally ill?”, 1 “definitely not” to 5 “definitely”) in the different lifestyles, two items with 5-point-likert scaled responses of the SHIP data collection were included. Furthermore, calculations described below, were stratified by median age and sex as well as having a mental illness. Latter operationalized as dichotomy of none and at least on self-reported mental illness symptom on the 12 items of the Composite International Diagnostic Screener (CID-S)^43^. All information are self-reported data.

**Figure 1:**
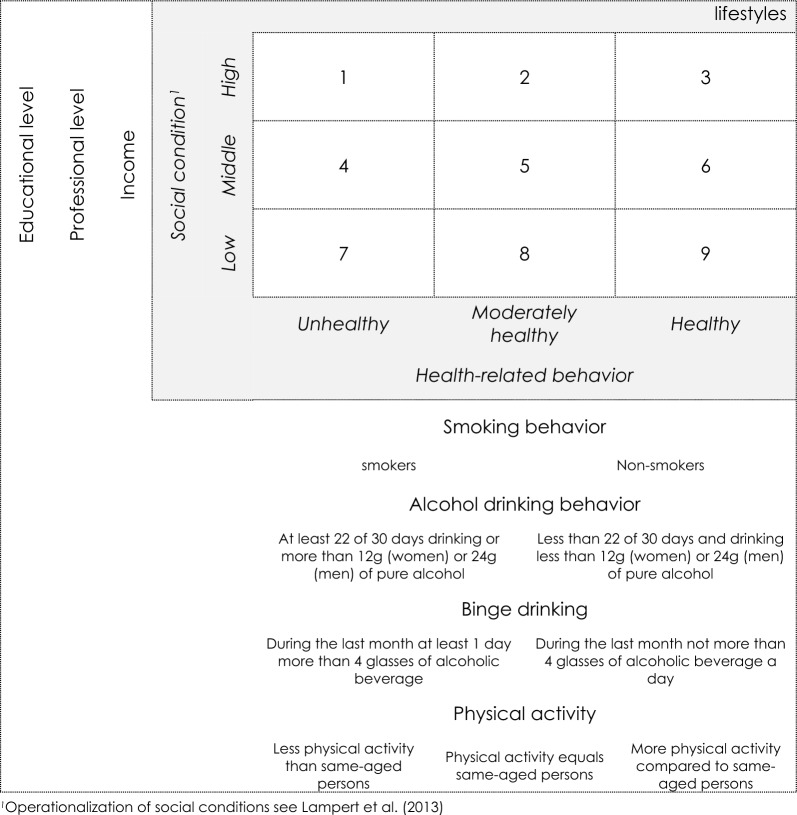
Simplified illustration of lifestyle operationalization (lifestyle 1: n = 64, 2: n = 208, 3: n = 36, 4: n = 207, 5: n = 599, 6: n = 115, 7: n = 92, 8: n = 189, 9: n = 53)

Figure 1 illustrates simplified operationalization for lifestyle-cluster.

### Statistical analysis

#### Description

*T* tests and Levene tests, ANOVAs to compare group means as well as Chi-squared tests to evaluate stochastically independence were calculated^41,44^.

#### Multilevel model

To analyze contextually dependent associations of shame (predictor) and help-seeking intentions (outcome) stepwise multilevel models were calculated^45,46^. Hierarchical linear model^47–49^ with homogeneous confirmatory groups^35^ of nine health-related lifestyles^12,36^ on level 2 (group level) focused on lifestyle-nested information for possible associations of shame and help-seeking intentions on level 1 (individual level). All models were stratified by dichotomous categories of sex, median-parted age and reported mental illness or not. For better interpretation (meaningful zero point, comparability of different scaled variables) data were standardized by group-mean-centering^50–53^. Regarding lifestyle clusters’ embedding in the complete sample, stepwise model calculations started with a null model (empty model) including help-seeking intentions varying between lifestyles. Intra-Class-Coefficient (ICC) indicated variance proportions explained by level-2-groups^54^. With the aim to predict willingness to seek help, the variable for shame was added to the model. First, fixed slope for different lifestyles in the random-intercept-model before random-intercept-random-slope-model was evaluated. The latter led to varying slopes for the association of shame and help-seeking intentions on individual as well as lifestyle level. After comparing estimations with restricted maximum likelihood (because of small numbers of level-2 groups), calculations were done via full maximum likelihood because of very small deviations between these estimates leading to interpretations of fixed as well as random effects^41,46,55,56^. All models and predictions were compared by Akaike’s as well as Bayesian Information Criteria^57,58^. Additionally model fit was analyzed by explained variance (overall R^2^ by Snijder and Bosker^59^, for each level referring to Raudenbush and Bryk^47,60^). Significance level was set to 95% (α = 0.05). Correction by multiple testing was not performed because of confirmatory hypotheses concerning stratified subgroups (for age and sex) and because multilevel model selection was not done by p-values^61^.

#### Software

All statistical operations were done with Stata SE 16.0^62^ with additional packages ‘multilevel tools’ to evaluate level-specific explained variance^63^, ‘spagplot’ to visualize level-specific graphs^64^ and ‘grc1leg’ to combine several graphs with one legend^65^.

### Ethics approval and consent to participate

The Study of Health in Pomerania (SHIP) was approved by the Ethics Committee at the University Medicine Greifswald, Germany (approval number BB 39/08)^31^ and is performed in accordance with the ethical standards laid down in the 1964 Declaration of Helsinki and its later amendments. All persons gave their informed consent prior to their inclusion in the study. Details that might disclose the identity of the subjects under study were omitted.

## Results

### Sample

The larger part of the SHIP-START-sample (n = 1630) were 54.1% women with a mean age of 59.8 (SD = 12.5) years. With a mean age of 61.5 (SD = 12.9) years, men were significant older (t(df = 1628) = 2.80, p = 0.007). 534 (32.76%) respondents reported a mental disorder. Study participants were grouped in nine lifestyle clusters regarding their socioeconomic status (ranging from low, middle to high) and their health-related behavior (healthy, moderately healthy and unhealthy). Supplement’s Table S1 presents descriptive results concerning lifestyle characteristics.

### Help-seeking intentions and shame concerning mental health in different lifestyles

#### Description

Shame scores were low with a mean of 1.75 (SD = 0.81, range: 1–5). The mean score for willingness to seek help was 3.93 (SD = 1.18, range: 1–5). Higher levels of shame were weakly associated with lower willingness to seek help (r = − 0.31, p < 0.001, adj. R^2^ = 0.044).

Variance analyses for lifestyle clusters showed no significant differences between feeling ashamed if they became mentally ill (Levene test: F(df = 8) = 0.71, p = 0.68; ANOVA: F(df = 8) = 0.84, p = 0.57). In contrast, there were differences between people in different lifestyles in the intention to seek professional help if they were mentally ill (Levene test: F(df = 8) = 5.37, p < 0.01; ANOVA: F(df = 8) = 2.28, p = 0.0203). Especially persons in lifestyles with high SES and moderate health behavior (lifestyle 2: M = 4.07, SD = 1.01) as well as middle-SES-moderately-healthy-lifestyle (lifestyle 5: M = 4.00, SD = 1.13) intended to seek help, whereas the high-SES-healthy-lifestyle (lifestyle 3: M = 3.5, SD = 1.23) or middle-SES-moderately-healthy-lifestyle (lifestyle 6: M = 3.69, SD = 1.28) showed less (but still distinct) intentions to seek help in case of a mental illness. Results are presented in Supplementary Table S1.

#### Nullmodel

Multilevel analyses considered hierarchical data structure with individuals’ help-seeking (level 1) nested in different lifestyles (level 2). Nullmodel with the outcome help-seeking intentions led to a between lifestyle-cluster variance close to zero with small differences between sex-stratified samples (men: ICC = 0.002; Standard-Error, SE = 0.006; women: ICC ≈ 0.000, SE = 0.000) but not for stratification by age and by reporting a mental illness (ICC ≈ 0.000, SE ≈ 0.000). Significant intercepts varied marginally between sex stratified samples (men: β_0_ = − 0.130, p < 0.001; women: β_0_ = − 0.120, p < 0.001). Age-stratification did not lead to significant intercepts (young: β_0_ = 0.060, p > 0.050; old: β_0_ = − 0.064, p > 0.050), similar to results for stratification by reporting a mental illness (no mental illness: β_0_ = − 0.001, p > 0.050; mental illness: β_0_ = 0.010, p > 0.050). Although ICC was low and therefore explanations by lifestyles were small, there is still an interest in having a further look on varying association by lifestyles. Therefore, shame was added to the model.

#### Random-intercept-model

Answering the question if the overall link of shame and help-seeking intentions varied between different lifestyles, random-intercept-models were calculated. Shame of a diagnosed mental illness as random intercept was added to the multilevel model (with fixed slope) and showed a significant association with the outcome help-seeking intention (β_1j_ = − 0.208, p < 0.001). Analyses concerning stratified samples led to stronger associations for men (β_1j_ = − 0.225, p < 0.001) than for women (β_1j_ = − 0.178, p < 0.001), for younger (β_1j_ = − 0.232, p < 0.001) than for older individuals (β_1j_ = − 0.184, p < 0.001), and for people without a mental illness (β_1j_ = − 0.281, p < 0.001) than people reporting a mental illness (β_1j_ = − 0.091, p < 0.05) in different lifestyles. Variances being explained by lifestyle differences were close to zero, but largest for men (u^2^_0j_ = 0.003). Overall, Snijders–Bosker-R^2^ was 0.043. Bryks–Raudenbush-R^2^ was 0.033 on level 2, lifestyles, and R^2^ was 0.043 on level 1 for participants.

#### Random-intercept-random-slope-model

Adding complexity to the model allowed a further look on varying associations of shame and willingness to seek help nested in lifestyles. Varying shame not only for intercept but also for the slopes, resulted in regression weights of shame and willingness to seek help with differences for men (β_1j_ = − 0.244, p < 0.001) compared to women (β_1j_ = − 0.178, p < 0.001) as well as younger (β_1j_ = − 0.238, p < 0.001) compared to older (β_1j_ = − 0.184, p < 0.001) people and people reporting no mental illness (β_1j_ = − 0.281, p < 0.001) compared to reporting mental illness (β_1j_ = − 0.117, p < 0.05) in different lifestyles. These slopes differed by lifestyles for male (*u*^*2*^_*1j*_ = 0.004) and younger (*u*^*2*^_*1j*_ = 0.017) participants and reporting mental illness (*u*^*2*^_*1j*_ = 0.010). Overall, Snijders–Bosker-R^2^ was 0.043. The hierarchical linear model without stratification explained 3.3% of variance by lifestyles and 4.3% for individuals by the mentioned connection of SES Index and health-related behavior.

**Figure 2:**
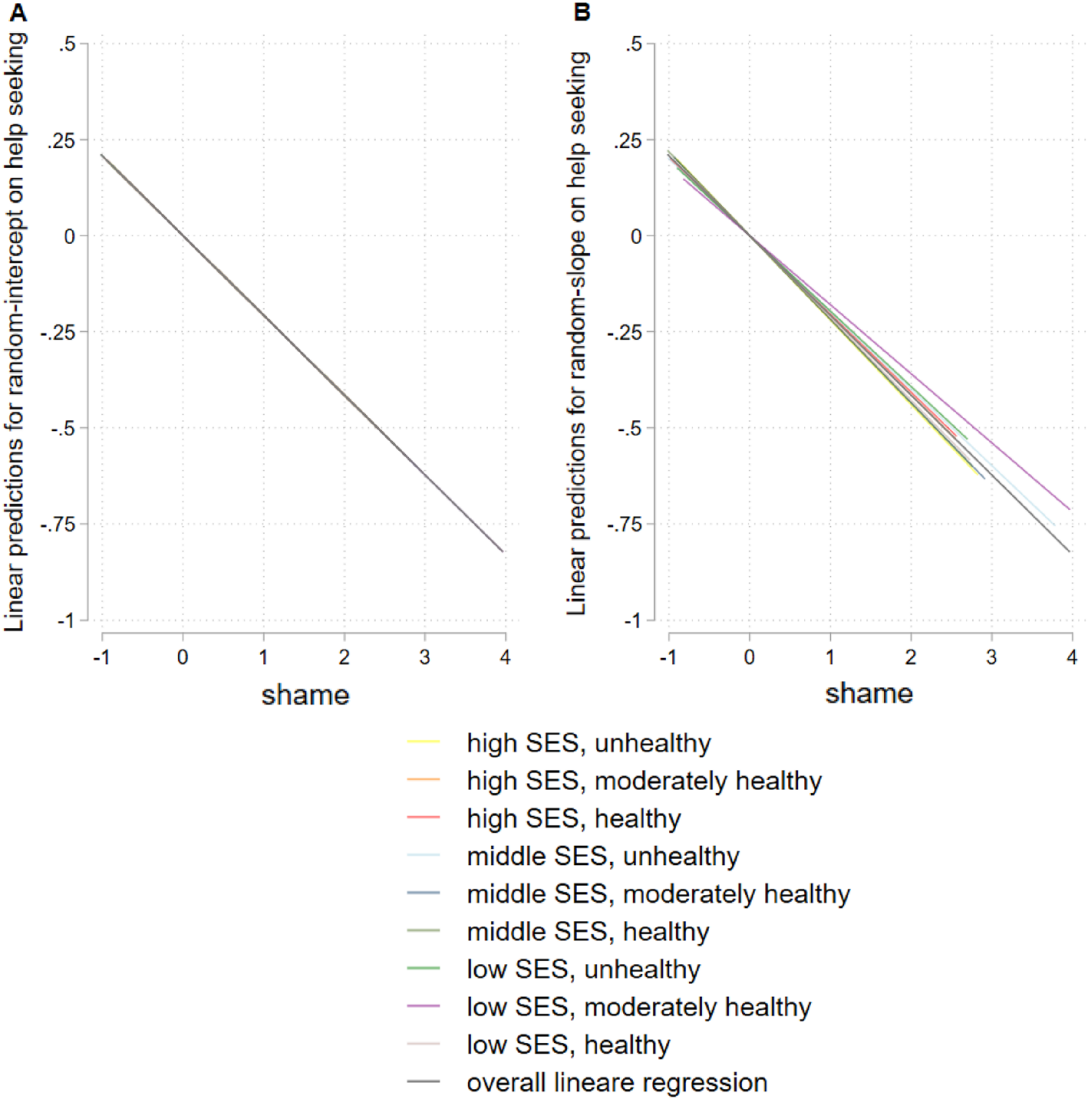
Hierarchical linear model comparison by linear predictions for random-intercept- (**A**) and random-intercept-random-slope-models (**B**) for linear prediction of shame and help seeking, overall, with survey participants on level 1 (n = 1630) and lifestyles on level 2 (m = 9), group mean standardized.

**Figure 3:**
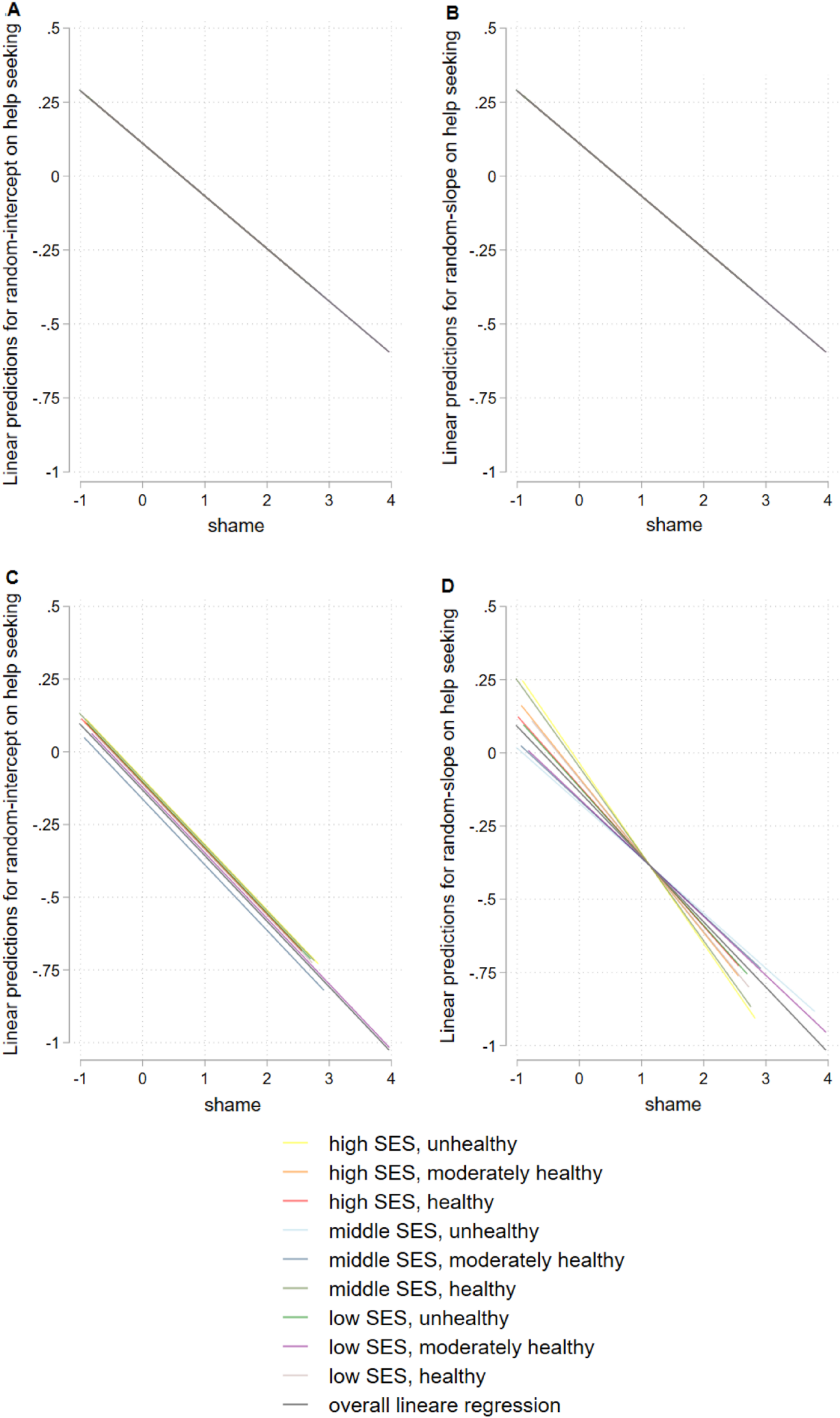
Hierarchical linear model comparison by linear predictions stratified by gender, random-intercept-model for women (**A**) and random-intercept-random-slope-model for women (**B**) random-intercept-model for men (**C**) and random-intercept-random-slope-model for men (**D**), with survey participants on level 1 (n = 1630) and lifestyles on level 2 (m = 9), group mean standardized.

**Figure 4:**
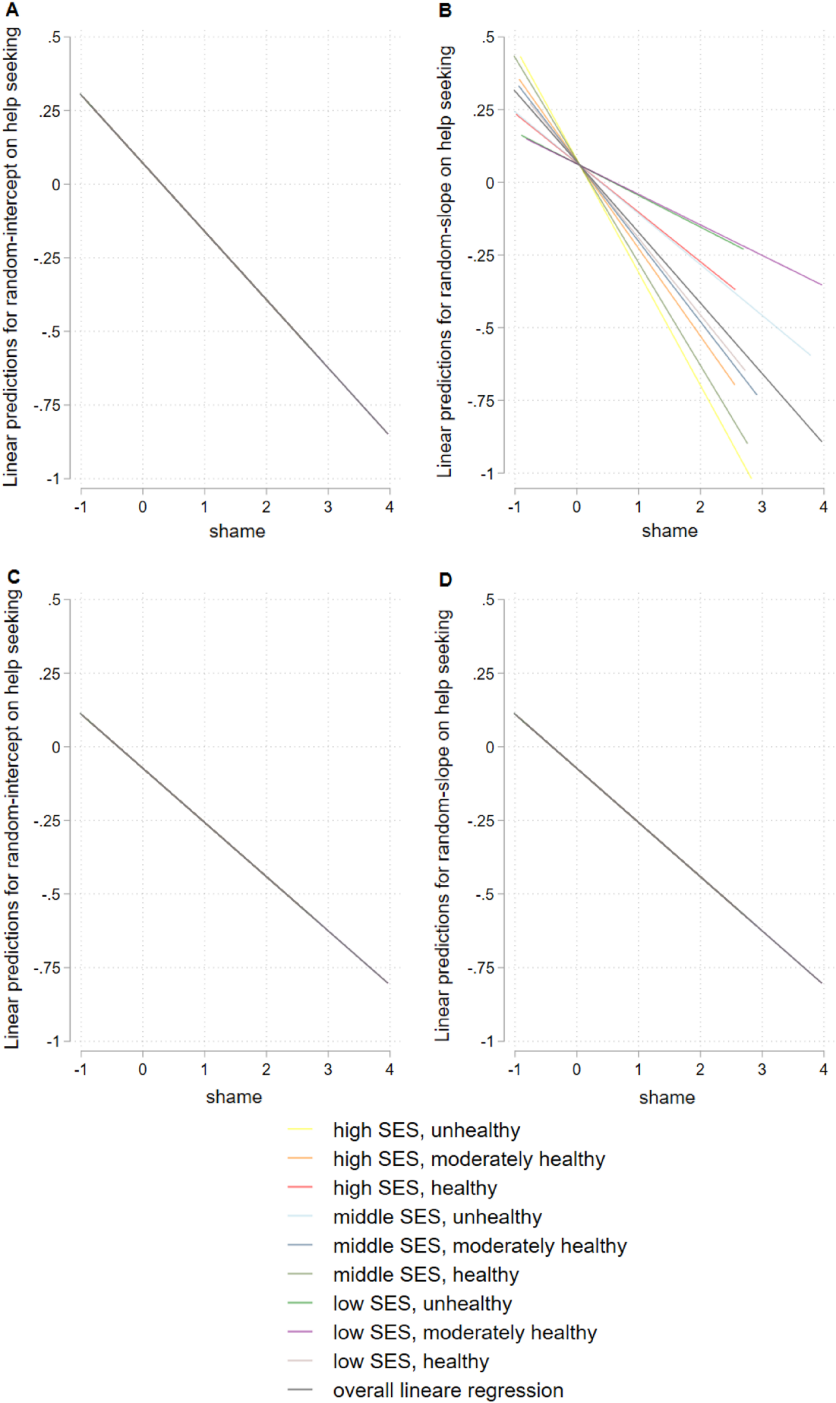
Hierarchical linear model comparison by linear predictions stratified by age, random-intercept-model for younger participants (age ≤ 61 y) (**A**) and random-intercept-random-slope-model for younger participants (age ≤ 61 y) (**B**) random-intercept-model for older participants (age > 61 y) (**C**) and random-intercept-random-slope-model for older participants (age > 61 y) (**D**), with survey participants on level 1 (n = 1630) and lifestyles on level 2 (m = 9), group mean standardized.

**Figure 5:**
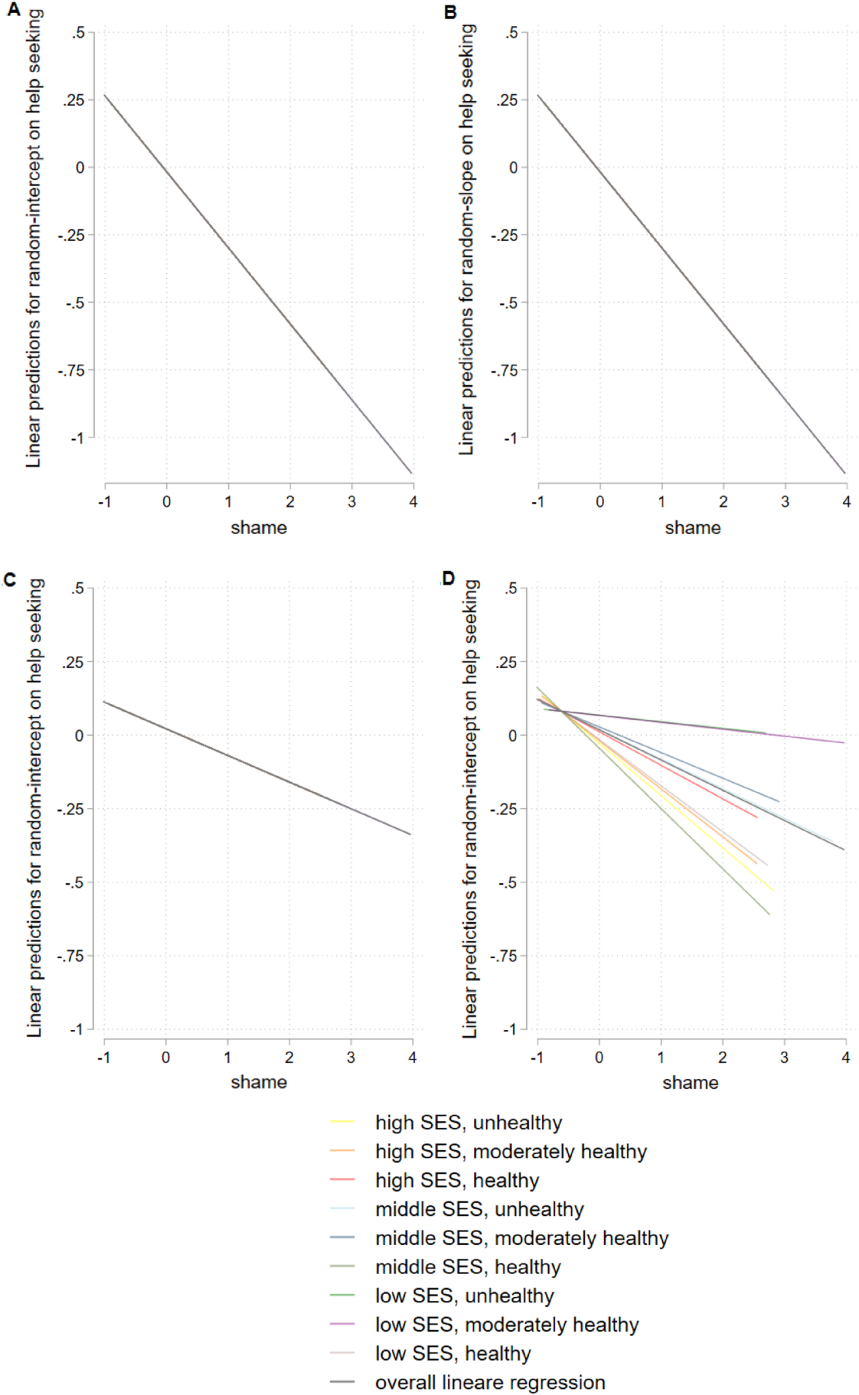
Hierarchical linear model comparison by linear predictions stratified by reported mental disorder, random-intercept-model for people without mental disorders (CID = 0) (**A**) and random-intercept-random-slope-model for people without mental disorders (CID = 0) (**B**) random-intercept-model people with mental disorders (CID > 0) (**C**) and random-intercept-random-slope-model for people with mental disorders (CID > 0) (**D**), with survey participants on level 1 (n = 1630) and lifestyles on level 2 (m = 9), group mean standardized.

Figure 2 shows results for hierarchical linear model for the total sample, Fig. 3 stratified for gender, Fig. 4 for age, and Fig. 5 for having a mental illness. Detailed results of hierarchical linear models are presented in Supplementary Table S2.1 for sex, Table S2.2 for age, and Table S2.3 for reported mental illness stratifications.

## Discussion

Overall, shame was moderately^66^ associated with help-seeking intentions in case of mental health problems for the whole sample. This is in line with past research (e.g.^67,68^). Confirming the hypothesis, clustered lifestyle characteristics showed very small context-effects, meaning that association of shame and willingness to seek professional help in case of mental illness varied little in lifestyles. Especially strong decreases in intentional help seeking for increased shame of a diagnosed mental illness were found in unhealthy behavior and high but also low SES. Middle-SES-and-unhealthy-behavior-lifestyle for men, and low-SES-and-moderately-healthy-behavior-lifestyle for younger participants as well as people reporting a mental illness showed weakest shame and help-seeking association compared to all other lifestyles. In these contexts, people considered to seek help even if they were ashamed of a diagnosed mental illness.

Golberstein et al.^69^ reported combinations of sociodemographic characteristics that led to higher stigma and varying help-seeking intentions. The present sample showed no varying shame in different lifestyles, which is in contrast to past research^15,70^. This might be due to the older sample which led to more homogenous findings as averaged shame for mental illness and experienced symptoms of mental disorders over life course reduced possible feelings of shame compared to younger individuals^69^. A lack of awareness, knowledge and communication about symptoms or not attributing them to mental illness in older people was accompanied by lower help seeking^71^. Homogenous findings might also relate to small number of confirmatory lifestyle-clusters (multilevel analyses with m = 9).

Reported mental illness, gender- and age-specific results were in line with past research for (self-)stigma^26,69^ or help-seeking research^5,8,10^. Gender-specific results correspond with past research^21,72^ and can be explained by women compared to men being socialized to take more care of themselves and others, and their experiences; for instance, they have more contact with doctors and are more likely to have health problems discovered earlier^73^. Homogenous association of shame and help-seeking in case of mental illness in different lifestyles of people reporting no mental illness might be due to lack of mental health literacy^74^.

Small associations of shame and help-seeking intentions in lifestyles with moderately healthy behaviors and middle SES might be a consequence of established public prevention strategies. Interventions reached a sample of averaged persons (expected to be a target group as huge as possible) instead of addressing certain barriers to health care system based on target groups or social clusters like lifestyles^9,29^. Due to the prevention paradox described by Bauer^75^ as well as Altgeld^76,77^ there is still a need of health prevention programs focusing on target groups besides people with high educational level and their perspectives (instead of academics’ views on marginalized groups and their problems). In this regard, the current findings are embedded in the context of the German health care system, characterized by structures of solidarity. Obligatory health insurances compensate for lifestyle-related inequalities like income^72^. Although these circumstances reduce some differences between people’s access to health care, there are still lifestyle-related backgrounds, socializations, experiences having influences on shame but especially on help-seeking intentions. These data might not be caught by the described system and possibly not comprehensively by the current data and should be addressed by future research.

Conceptualizing social structures by described health-related lifestyles helped to identify target groups: men and younger people with unhealthy lifestyles and different socioeconomic status should be particularly addressed by prevention programs. Therefore, lifestyle clusters were a useful tool for evaluating associations of stigma and willingness to seek help besides operationalized milieus or classes^15^. Because lifestyles refer to persons’ behavior being more flexible compared to social context with income, they are a modern approach to evaluate possible target groups^16^ and also a useful tool for further research question in public health being related to disease management, contacts with health services^29,39^ or to draw conclusions on attitudes, openness, health literacy, stereotypes, stigmata, intersectionality etc.^15^.

### Future directions

In sum shame is a barrier in seeking help^10,11^. Embedded in the confirmatory concept of lifestyles including the combination of health-related behavior and sociodemographic characteristics, the present operationalization might not cover all aspects explaining different associations in population’s shame and help-seeking intentions. Besides institutionalized enabled access to the health care system, there is still the need to sufficiently address different target groups to overcome individual unhealthy behaviors, attitudes and habits including shame of a diagnosed mental illness. Targeting social disparities, reaching a variety of marginalized groups, consider people’s plans and reality of life (values, goals related to stages of life among others) should be future directions for anti-stigma and prevention programs for mental health problems^76,78^. Further, mental health status^21,79^, psychopathology^8,80^, mental health literacy^21,26,74^, ethnic minorities^8^, migration^13^, intersectionality^27,81^ play important roles and should be addressed by future research. Overall, to reduce shame and stigma for mental illness to increase help-seeking intentions in the mentioned target groups suggested strategies include prevention^29,77^, education or communication^81,82^.

## Strengths and limitations

### Data collection

The Study of Health in Pomerania is a population-based project consisting of the three cohorts SHIP-START, SHIP-TREND and SHIP-NEXT^31^. Though, northeast Germany equals more rural areas and more women were part of the population limiting representativity^7,26^. Possible changes in educational or professional status over time were disregarded because of the high aged population. Arguing that help-seeking referred to intentions and was asked hypothetically while health-behavior based on retrospective questions seems useful to disprove the confounding of help-seeking and health-behavior. As current results implicate that health related lifestyles play an important role in stigma research, future investigation might include more complex approaches like clustering additionally sleeping and eating habits. Further, the current analyses included only self-rated information. Thinking about possible social desirability^45^, future directions might also take objective health-related data^39^ into consideration, like laboratory values, physicians’ ratings or diagnoses, prevention recommendations or geographical features (e.g. density of psychotherapists)^83^. Weighting aspects of health-related lifestyles might be useful for differences in risks for several diseases^39^. Besides, physical activity was not separated for activity during leisure time or being work-related. This might lead to participants’ misunderstandings and possible underestimation as individuals not considering work-related physical activity. Additionally, our sample did not reveal any remarkable differences in shame and help-seeking between former and current smokers. Nevertheless it is reasonable that former smokers might have an increased health literacy or awareness for a healthy lifestyle in course of their decision not to smoke anymore^84^ which is associated with shame and help-seeking behavior^21^.

Additional variables and interrelations not being part of the present data collection should be considered: Reduced willingness to seek help in different lifestyles might be caused by individuals being afraid of bad treatments^85^, gender role conflicts especially for men^86,87^, differentiation between varying mental illness’ and inherent symptoms^7,79^ or mental health literacy^21,67^. The mentioned barriers might be lowered by positively experienced primary or secondary mental health care^10,21,88^, social support^8,89^ or addressing particularly target groups at risk^3,69^. Positive experiences with health care, e.g. avoiding long periods of waiting for professionals or realistic expectations about the therapy’s outcome promoted help seeking in case of mental health problems^10^. Beyond health behavior and socioeconomic status, destigmatization of mental illness in lifestyles may also be part of more complex interrelation with pop culture^90^, values, attitudes^91^, and so on.

### Methodological aspects

The present paper aimed to meet the need for mental health focus, integrating shame, help-seeking intentions and health-related lifestyles to overcome one-dimensional sociodemographic approaches. Confirmatory lifestyle clusters are always artificial, simplified definitions of combination of characteristics while modern society based on fluid boundaries and changeable habits especially in crises^16^. On the other hand, compared to sample specific, statistical cluster analyses, these confirmatory lifestyles could be analyzed across time and samples. By investigating associations of shame and help-seeking intentions in different lifestyles, we could not conclude causal inference^41^. Future research should consider long-term data collections evaluating societal changes, predicting fitting lifestyle cluster, their features in relation to shame of being mentally ill and willingness to seek help. Multilevel model’s total variance was marginally explained by lifestyles. This might imply more simple statistical techniques^92^ but seemed misleading for identifying possible marginalized target groups being undermined or not viewed differentiated. Moreover, less clusters with different numbers of individuals within groups might have an influence on sufficient power and estimates even there is a lack of general recommendations concerning sample sizes for multilevel models^42,93^.

## Conclusion

Instead of focusing on averages and norms, groups with markedly high levels of shame and low help-seeking intentions in case of mental illness as well as possible causes based on values, attitudes and behaviors can be addressed. Therefore, lifestyles are appropriate and potentially helpful tools for public health research to identify marginalized groups with different socioeconomic status but unhealthy behaviors, with high stigma and low intentions to seek help. Even in reducing complexity and artificial distinction between social groups being fluctuating and diverse, various analyses concerning health-related behavior, health literacy, evaluation of destigmatization and prevention programs are possible.

### Supplementary Information

Supplementary Tables.

## Data Availability

For data requests, please contact the corresponding author, Claudia Helmert, claudia.helmert@medizin.uni-leipzig.de.

## Abbreviations

CWC: Centering within cluster
ICC: Intra class coefficient
M: Mean
SD: Standard deviation
SE: Standard error
SES: Socio-economic status
SHIP: Study of Health in Pomerania
