# Supplementary material for: An explorative cross-sectional analysis of mental health shame and help-seeking intentions in different lifestyles
Source: Sci Rep. 2023 Jul 4;13:10825. doi: 10.1038/s41598-023-37955-8 (PMC10319876; doi:10.1038/s41598-023-37955-8)
Supplement: Supplementary file 1 — Supplementary Tables. [file 41598_2023_37955_MOESM1_ESM.pdf]

An Explorative Cross-Sectional Analysis of Mental-Health Shame and Help-Seeking-Intention in different Lifestyles

Social Psychiatry und Psychiatric Epidemiology

Claudia Helmert*[1], Toni Fleischer[1], Sven Speerforck[1], Christine Ulke[1], Laura Altweck[2], Stefanie Hahm[2], Holger Mühlan[2], Silke Schmidt[2], Hans J. Grabe[3], Henry Völzke[4], Georg Schomerus[1]
[1]Department of Psychiatry and Psychotherapy, University of Leipzig Medical Center, Leipzig, Germany
[2]Department of Health and Prevention, University of Greifswald, Greifswald, Germany
[3]Department of Psychiatry and Psychotherapy, Greifswald University, Medical Center, Greifswald, Germany
[4]Institute for Community Medicine, Greifswald University, Medical Center, Greifswald, Germany
*Corresponding Author: Claudia Helmert, claudia.helmert@medizin.uni-leipzig.de

Supplementary Information

Table S1: Sample characteristics for each lifestyle, frequencies by column (except for the distribution of lifestyle), the column „missings“ includes missing values for survey participants being not assigned to a lifestyle because of missing values in at least one domain 2

Table S2.1: Hierarchical linear model comparison by sex of group mean standardized results with outcome help-seeking intentions (full-maximum-likelihood-estimation; index i: level 1-unit – survey participants, n=1630; index j: level-2-unit – lifestyles, m=9) 9

Table S2.2: Hierarchical linear model comparison by age of group mean standardized results with outcome help-seeking intentions (full-maximum-likelihood-estimation; index i: level 1-unit – survey participants, n=1630; index j: level-2-unit – lifestyles, m=9) 10

Table S2.3: Hierarchical linear model comparison by having a mental illness or not of group mean standardized results with outcome help-seeking intentions (full-maximum-likelihood-estimation; index i: level 1-unit – survey participants, n=1630; index j: level-2-unit – lifestyles, m=9) 11

An Explorative Cross-Sectional Analysis of Mental-Health Shame and Help-Seeking-Intention in different Lifestyles

Social Psychiatry und Psychiatric Epidemiology

Claudia Helmert*[1], Toni Fleischer[1], Sven Speerforck[1], Christine Ulke[1], Laura Altweck[2], Stefanie Hahm[2], Holger Mühlan[2], Silke Schmidt[2], Hans J. Grabe[3], Henry Völzke[4], Georg Schomerus[1]
[1]Department of Psychiatry and Psychotherapy, University of Leipzig Medical Center, Leipzig, Germany
[2]Department of Health and Prevention, University of Greifswald, Greifswald, Germany
[3]Department of Psychiatry and Psychotherapy, Greifswald University, Medical Center, Greifswald, Germany
[4]Institute for Community Medicine, Greifswald University, Medical Center, Greifswald, Germany
*Corresponding Author: Claudia Helmert, claudia.helmert@medizin.uni-leipzig.de

Table S1: Sample characteristics for each lifestyle, frequencies by column (except for the distribution of lifestyle), the column „missings" includes missing values for survey participants being not assigned to a lifestyle because of missing values in at least one domain

					Lifestyles [a]								
	Descriptives		*Total*	*Missings*	1	2	3	4	5	6	7	8	9
		N	1630	67	65	220	23	208	623	90	95	192	47
Variables		%		4.11	3.99	13.50	1.41	12.76	38.22	5.52	5.83	11.78	2.88
Sex	Men	N	749	33	51	115	10	119	238	34	60	76	13
		%	45.95	49.25	78.46	52.27	43.48	57.21	38.20	37.78	63.16	39.58	27.66
	Women	N	881	34	14	105	13	89	385	56	35	116	34
		%	54.05	50.75	21.54	47.73	56.52	42.79	61.80	62.22	36.84	60.42	72.34
	Missings	N	0	0	0	0	0	0	0	0	0	0	0
		%	0	0	0	0	0	0	0	0	0	0	0
Age		*Median*			*57*	*58*	*61*	*53*	*63*	*70*	*58*	*64*	*71*
		M	60.60	63.54	55.63	58.98	57.43	53.77	61.92	65.67	57.56	63.42	69.98
		SD	12.71	14.81	11.42	12.52	14.19	10.46	12.38	13.67	10.00	12.37	10.92
		Range	36-93	37-91	37-82	37-91	37-81	36-83	36-93	37-90	37-80	36-89	38-92
		Missings	0	0	0	0	0	0	0	0	0	0	0
Having a mental illness	No (CID=0)	N	1080	31	48	131	25	140	414	85	55	116	35
		%	66.26	46.27	75.00	62.98	69.44	67.63	69.12	73.91	59.78	61.38	66.04
	Yes (CID>0)	N	534	26	16	77	10	67	183	28	36	73	18
		%	32.76	38.81	25.00	37.02	27.78	32.37	30.55	24.35	39.13	38.62	33.96
	Missings	N	16	10	0	0	1	0	2	2	1	0	0
		%	0.98	14.93	0	0	2.78	0	0.33	1.74	1.09	0	0
Would you feel ashamed if you were mentally ill?		Median	2	1	2	2	2	2	2	2	2	1-2	1
		M	1.75	1.65	1.73	1.80	1.83	1.85	1.73	1.81	1.75	1.69	1.65
		SD	0.81	0.79	0.80	0.86	0.85	0.83	0.78	0.79	0.83	0.84	0.86
		Range	1-5	1-4	1-4	1-4	1-4	1-5	1-4	1-4	1-4	1-5	1-4
		Missings	4										
	Not at all	N	717	34	28	96	9	78	270	37	44	96	25
		%	43.99	50.75	43.08	43.64	39.13	37.50	43.34	41.11	46.32	50.00	53.19
	A little	N	659	23	29	80	12	97	266	36	36	68	12
		%	40.43	34.33	44.62	36.36	52.17	46.63	42.70	40.00	37.89	35.42	25.53
	Moderately	N	187	7	5	32	2	22	67	13	11	21	7
		%	11.47	10.45	7.69	14.55	9.70	10.58	10.75	14.44	11.58	10.94	14.89
	Much	N	60	2	3	12	0	10	19	4	4	4	2
		%	3.68	2.99	4.62	5.45	0	4.81	3.05	4.44	4.21	2.08	4.26
	Very much	N	3	0	0	0	0	1	0	0	0	2	0
		%	0.18	0	0	0	0	0.48	0	0	0	1.04	0
	Missings	N	4	1	0	0	0	0	1	0	0	1	1
		%	0.24	1.49	0	0	0	0	0.16	0	0	0.52	2.13

Claudia Helmert[*1], Toni Fleischer[1], Sven Speerforck[1], Christine Ulke[1], Laura Altweck[2], Stefanie Hahm[2], Holger Mühlan[2], Silke Schmidt[2], Hans J. Grabe[3], Henry Völzke[4], Georg Schomerus[1]
[1]Department of Psychiatry and Psychotherapy, University of Leipzig Medical Center, Leipzig, Germany
[2]Department of Health and Prevention, University of Greifswald, Greifswald, Germany
[3]Department of Psychiatry and Psychotherapy, Greifswald University, Medical Center, Greifswald, Germany
[4]Institute for Community Medicine, Greifswald University, Medical Center, Greifswald, Germany
[*]Corresponding Author: Claudia Helmert, claudia.helmert@medizin.uni-leipzig.de

					Lifestyles [a]								
	Descriptives		*Total*	*Missings*	1	2	3	4	5	6	7	8	9
		N	1630	67	65	220	23	208	623	90	95	192	47
Variables		%		4.11	3.99	13.50	1.41	12.76	38.22	5.52	5.83	11.78	2.88
Would you seek professional help if you feel depressed for a long time or if you had other mental problems?		Median	4	4	4	4	4	4	4	4	4	5	5
		M	3.93	3.82	3.90	4.07	3.5	3.87	4.00	3.69	3.70	3.98	3.98
		SD	1.18	1.40	1.16	1.01	1.23	1.17	1.13	1.28	1.35	1.26	1.24
		Range	1-5	1-5	1-5	1-5	1-5	1-5	1-5	1-5	1-5	1-5	1-5
		Missings	5										
	Definitely not	N	57		2	1	2	7	17	6	6	9	1
		%	3.51		3.08	0.45	8.70	3.37	2.73	6.67	6.32	4.69	2.13
	2	N	167		6	19	3	23	52	14	14	20	8
		%	10.28		9.23	8.64	13.04	11.06	8.35	15.56	14.74	10.42	17.02
	3	N	353		15	49	5	44	138	22	24	37	9
		%	21.72		23.08	22.27	21.74	21.15	22.15	24.44	25.56	19.27	19.15
	4	N	307		13	54	7	48	125	16	9	22	4
		%	18.89		20.00	24.55	30.43	23.08	20.06	17.78	9.47	11.46	8.51
	Definitely	N	741		28	97	6	86	291	32	42	103	24
		%	45.60		43.08	44.09	26.07	41.35	46.71	35.56	44.21	53.65	51.06
	Missings	N	5		1	0	0	0	0	0	0	1	1
		%	0.30		1.54	0	0	0	0	0	0	0.52	2.13
Association of shame (Predictor) and help-seeking (outcome) (without recognizing hierarchical data structure)	b0	Coeff.	4.46		4.98	4.52	3.87	4.31	4.58	4.59	3.97	4.11	4.83
		P	*<0.001*		*<0.001*	*<0.001*	*<0.001*	*<0.001*	*<0.001*	*<0.001*	*<0.001*	*<0.001*	*<0.001*
	r	Coeff.	-.31		-.61	-.25	-.2	-.24	-.34	-.50	-.16	-.08	-.51
		p	<0.001		0.001	0.002	0.42	0.01	<0.001	0.001	0.35	0.48	0.01
	R^2 (adj.)		0.04		0.17	0.04	-0.01	0.02	0.05	0.09	-0.002	-0.003	0.11
Endogenous : SES & Health status													
SES													
Income	Less than 500 €	N	33	4	0	0	0	0	0	0	7	21	1
		%	2.02	5.97	0	0	0	0	0	0	7.37	10.94	2.13
	500-900 €	N	107	2	0	0	0	0	5	1	29	60	10
		%	6.56	2.99	0	0	0	0	0.80	1.11	30.53	31.25	21.28
	900-1300 €	N	158	8	0	0	0	10	52	2	19	50	17
		%	9.69	11.94	0	0	0	4.81	8.35	2.22	20.00	26.04	36.17
	1300-1800€	N	318	9	1	7	3	30	140	35	31	50	12
		%	19.51	13.43	1.54	3.18	13.04	14.42	22.47	38.89	32.63	26.04	25.53
	1800-2300€	N	274	4	1	19	2	64	157	17	3	5	2
		%	16.81	5.97	1.54	8.64	8.70	30.77	25.20	18.89	3.16	2.60	4.26
	2300-2800€	N	215	3	9	37	2	35	115	13	0	1	0
		%	13.19	4.48	13.85	16.82	8.70	16.83	18.46	14.44	0	0.52	0

An Explorative Cross-Sectional Analysis of Mental-Health Shame and Help-Seeking-Intention in different Lifestyles

Social Psychiatry und Psychiatric Epidemiology

Claudia Helmert[*1], Toni Fleischer[1], Sven Speerforck[1], Christine Ulke[1], Laura Altweck[2], Stefanie Hahm[2], Holger Mühlan[2], Silke Schmidt[2], Hans J. Grabe[3], Henry Völzke[4], Georg Schomerus[1]

[1]Department of Psychiatry and Psychotherapy, University of Leipzig Medical Center, Leipzig, Germany

[2]Department of Health and Prevention, University of Greifswald, Greifswald, Germany

[3]Department of Psychiatry and Psychotherapy, Greifswald University, Medical Center, Greifswald, Germany

[4]Institute for Community Medicine, Greifswald University, Medical Center, Greifswald, Germany

[*]Corresponding Author: Claudia Helmert, claudia.helmert@medizin.uni-leipzig.de

					Lifestyles [a]								
Variables	Descriptives		*Total*	*Missings*	1	2	3	4	5	6	7	8	9
		N	1630	67	65	220	23	208	623	90	95	192	47
		%		4.11	3.99	13.50	1.41	12.76	38.22	5.52	5.83	11.78	2.88
	2800-3300€	N	165	4	14	42	5	30	63	7	0	0	0
		%	10.12	5.97	21.54	19.09	21.74	14.42	10.11	7.78	0	0	0
	3300-3800€	N	101	6	11	24	5	16	33	6	0	0	0
		%	6.20	8.96	16.92	10.91	21.74	7.69	5.30	6.67	0	0	0
	3800-4300€	N	73	5	8	30	0	12	15	3	0	0	0
		%	4.48	7.46	12.31	13.64	0	5.77	2.41	3.33	0	0	0
	4300-4800€	N	45	1	9	13	3	4	14	2	0	0	0
		%	2.76	1.49	13.85	5.91	8.70	1.92	2.25	2.22	0	0	0
	4800-5300€	N	35	1	4	18	0	4	8	0	0	0	0
		%	2.15	1.49	6.15	8.18	0	1.92	1.28	0	0	0	0
	5300-5800€	N	15	0	3	8	1	1	2	0	0	0	0
		%	0.92	0	4.62	3.64	4.35	0.48	0.32	0	0	0	0
	5800-6300€	N	10	0	2	4	1	1	2	0	0	0	0
		%	0.61	0	3.08	1.82	4.35	0.48	0.32	0	0	0	0
	6300-7800€	N	13	2	1	5	2	0	3	0	0	0	0
		%	0.80	2.99	1.54	2.27	8.70	0	0.48	0	0	0	0
	More than 7800€	N	9	0	1	6	0	0	1	1	0	0	0
		%	0.55	0	1.54	2.73	0	0	0.16	1.11	0	0	0
	Missings	N	59	18	1	7	0	1	13	0	6	5	5
		%	1.04	27.06	1.54	3.18	0	0.48	2.09	0	6.32	2.60	10.64
Profession	Self-employed farmer	N	2	0	0	0	0	0	1	0	0	1	0
		%	0.12	0	0	0	0	0	0.16	0	0	0.52	0
	Academic freelancer	N	29	1	4	20	2	0	2	0	0	0	0
		%	1.78	1.49	6.15	9.09	8.70	0	0.32	0	0	0	0
	Other self-employed up to 9 employees	N	82	2	12	23	2	11	26	2	3	1	0
		%	5.03	2.99	18.46	10.45	8.70	5.29	4.17	2.22	3.16	0.52	0
	Other self-employed more than 9 employees	N	5	0	0	1	0	0	3	1	0	0	0
		%	0.31	0	0	0.45	0	0	0.48	1.11	0	0	0
	Civil servants, judge, professional soldier (ordinary civil service)	N	5	1	0	0	0	2	2	0	0	0	0
		%	0.31	1.49	0	0	0	0.96	0.32	0	0	0	0
	Civil servants, judge, professional soldier (middle civil service)	N	21	0	3	3	0	5	9	1	0	0	0
		%	1.29	0	4.62	1.36	0	2.40	1.44	1.11	0	0	0

Claudia Helmert*[1], Toni Fleischer[1], Sven Speerforck[1], Christine Ulke[1], Laura Altweck[2], Stefanie Hahm[2], Holger Mühlan[2], Silke Schmidt[2], Hans J. Grabe[3], Henry Völzke[4], Georg Schomerus[1]
[1]Department of Psychiatry and Psychotherapy, University of Leipzig Medical Center, Leipzig, Germany
[2]Department of Health and Prevention, University of Greifswald, Greifswald, Germany
[3]Department of Psychiatry and Psychotherapy, Greifswald University, Medical Center, Greifswald, Germany
[4]Institute for Community Medicine, Greifswald University, Medical Center, Greifswald, Germany
*Corresponding Author: Claudia Helmert, claudia.helmert@medizin.uni-leipzig.de

Variables					Lifestyles [a]								
	Descriptives		*Total*	*Missings*	1	2	3	4	5	6	7	8	9
		N	1630	67	65	220	23	208	623	90	95	192	47
		%		4.11	3.99	13.50	1.41	12.76	38.22	5.52	5.83	11.78	2.88
	Civil servants, judge, professional soldier (senior civil service)	N	17	2	4	9	0	0	1	1	0	0	0
		%	1.04	2.99	6.15	4.09	0	0	0.16	1.11	0	0	0
	Civil servants, judge, professional soldier (higher civil service)	N	6	0	2	4	0	0	0	0	0	0	0
		%	0.37	0	3.08	1.82	0	0	0	0	0	0	0
	Employee with simple tasks	N	107	3	0	0	0	11	33	2	15	36	7
		%	6.56	4.48	0	0	0	5.29	5.30	2.22	15.79	18.75	14.89
	Employee with qualified tasks	N	422	20	3	16	3	64	226	35	10	29	16
		%	25.89	29.85	4.62	7.27	13.04	30.77	36.28	38.89	10.53	15.10	34.04
	Employee with independent task completion / responsible position	N	470	13	26	105	14	51	216	28	1	14	2
		%	28.83	19.40	40.00	47.73	60.87	24.52	34.67	31.11	1.05	7.29	4.26
	Employees with management duties	N	97	3	10	37	2	3	32	7	0	3	0
		%	5.95	4.48	15.38	16.82	8.70	1.44	5.14	7.78	0	1.56	0
	Workers (unskilled)	N	39	3	0	0	0	2	3	0	7	20	4
		%	2.39	4.48	0	0	0	0.96	0.48	0	7.37	10.42	8.51
	Workers (semi-skilled)	N	45	3	0	0	0	5	1	1	10	21	4
		%	2.76	4.48	0	0	0	2.40	0.16	1.11	10.53	10.94	8.51
	Workers (skilled)	N	230	14	0	0	0	48	53	7	43	54	11
		%	14.11	20.90	0	0	0	23.08	8.51	7.78	45.26	28.13	23.40
	Workers (master, polisher, brigadier)	N	45	2	0	0	0	6	14	4	5	11	3
		%	2.76	2.99	0	0	0	2.88	2.25	4.44	5.26	5.73	6.38
	Missings	N	8	0	1	2	0	0	1	1	1	2	0
		%	0.49	0	1.54	0.91	0	0	0.16	1.11	1.05	1.04	0
Education level	No graduation (and I no longer go to school)	N	19	1	0	0	0	1	3	2	2	6	4
		%	1.17	1.49	0	0	0	0.48	0.48	2.22	2.11	3.13	8.51
	Elementary / secondary school / polytechnical high school 8th/9th grade	N	398	24	0	1	0	25	146	26	42	101	33
		%	24.42	35.82	0	0.45	0	12.02	23.43	28.89	44.21	52.60	70.21
	Secondary school / polytechnical high school	N	841	31	16	31	1	171	402	52	47	80	10
		%	51.60	46.27	24.62	14.09	4.35	82.21	64.53	57.78	49.47	41.67	12.28

An Explorative Cross-Sectional Analysis of Mental-Health Shame and Help-Seeking-Intention in different Lifestyles
Social Psychiatry und Psychiatric Epidemiology
Claudia Helmert[*1], Toni Fleischer[1], Sven Speerforck[1], Christine Ulke[1], Laura Altweck[2], Stefanie Hahm[2], Holger Mühlan[2], Silke Schmidt[2], Hans J. Grabe[3], Henry Völzke[4], Georg Schomerus[1]
[1]Department of Psychiatry and Psychotherapy, University of Leipzig Medical Center, Leipzig, Germany
[2]Department of Health and Prevention, University of Greifswald, Greifswald, Germany
[3]Department of Psychiatry and Psychotherapy, Greifswald University, Medical Center, Greifswald, Germany
[4]Institute for Community Medicine, Greifswald University, Medical Center, Greifswald, Germany
[*]Corresponding Author: Claudia Helmert, claudia.helmert@medizin.uni-leipzig.de

					Lifestyles [a]								
	Descriptives		*Total*	*Missings*	1	2	3	4	5	6	7	8	9
		N	1630	67	65	220	23	208	623	90	95	192	47
Variables		%		4.11	3.99	13.50	1.41	12.76	38.22	5.52	5.83	11.78	2.88
	Technical college graduation	N	34	2	4	10	4	2	11	0	1	0	0
		%	2.09	2.99	6.15	4.55	17.39	0.96	1.77	0	1.05	0	0
	(Extended) High school graduation	N	330	8	45	177	17	9	59	9	2	4	0
		%	20.25	11.94	69.23	80.45	73.91	4.33	9.47	10.00	2.11	2.08	0
	Other school diploma	N	7	1	0	1	1	0	2	0	1	1	0
		%	0.43	1.49	0	0.45	4.35	0	0.32	0	1.05	0.52	0
	Missings	N	1	0	0	0	0	0	0	1	0	0	0
		%	0.06	0	0	0	0	0	0	1.11	0	0	0
Profession level	In vocational training / pupil / student	N	8	0	2	1	0	1	2	0	1	1	0
		%	0.49	0	3.08	0.45	0	0.48	0.32	0	1.05	0.52	0
	No educational qualification / part-skilled worker	N	50	3	0	0	0	0	3	1	8	24	11
		%	3.07	4.48	0	0	0	0	0.48	1.11	8.42	12.50	23.40
	Skilled worker / apprenticeship with degree	N	735	39	10	20	4	112	280	37	73	133	27
		%	45.09	58.21	15.38	9.09	17.39	54.33	44.94	41.11	76.84	69.27	57.45
	Apprenticeship master / technician	N	131	5	2	8	1	23	64	8	8	9	3
		%	8.04	7.46	3.08	3.64	4.35	11.06	10.27	8.89	8.42	4.69	6.38
	Professional / business school	N	45	2	3	0	0	9	21	4	1	5	6
		%	2.76	2.99	4.62	0	0	4.33	3.37	4.44	1.05	2.60	12.77
	Technical (high) school degree	N	408	9	23	48	5	55	210	32	3	17	0
		%	25.03	13.43	35.38	21.82	21.74	26.44	33.71	35.56	3.16	8.85	0
	High school degree	N	252	8	25	143	12	8	43	7	1	3	0
		%	15.46	13.43	38.46	65.00	56.52	3.85	6.90	7.78	1.05	1.56	0
	Missings	N	1	0	0	0	0	0	0	1	0	0	0
		%	0.06	0	0	0	0	0	0	1.11	0	0	0
Health status													
Smokers	No	N	1330	18	42	215	23	103	580	90	45	167	47
		%	81.60	26.87	64.62	97.73	100	49.52	93.10	100	47.37	86.98	100
	Yes	N	285	34	23	5	0	105	43	0	50	25	0
		%	17.48	50.75	35.38	2.27	0	50.48	6.90	0	52.63	13.02	0
	Missings	N	15	15	0	0	0	0	0	0	0	0	0
		%	0.92	22.38	0	0	0	0	0	0	0	0	0
Alcohol (including people who are not drinking „0“ part of the average)	Binge drinking days (more than 5 glasses of alcohol) during the last	M	1.02	0.65	2.54	0.44	0	2.83	0.26	0	3.66	0.28	0

An Explorative Cross-Sectional Analysis of Mental-Health Shame and Help-Seeking-Intention in different Lifestyles
Social Psychiatry und Psychiatric Epidemiology
Claudia Helmert*[1], Toni Fleischer[1], Sven Speerforck[1], Christine Ulke[1], Laura Altweck[2], Stefanie Hahm[2], Holger Mühlan[2], Silke Schmidt[2], Hans J. Grabe[3], Henry Völzke[4], Georg Schomerus[1]
[1]Department of Psychiatry and Psychotherapy, University of Leipzig Medical Center, Leipzig, Germany
[2]Department of Health and Prevention, University of Greifswald, Greifswald, Germany
[3]Department of Psychiatry and Psychotherapy, Greifswald University, Medical Center, Greifswald, Germany
[4]Institute for Community Medicine, Greifswald University, Medical Center, Greifswald, Germany
*Corresponding Author: Claudia Helmert, claudia.helmert@medizin.uni-leipzig.de

					Lifestyles [a]								
	Descriptives		*Total*	*Missings*	1	2	3	4	5	6	7	8	9
		N	1630	67	65	220	23	208	623	90	95	192	47
Variables		%		4.11	3.99	13.50	1.41	12.76	38.22	5.52	5.83	11.78	2.88
	Month	SD	3.02		4.46	1.48	0	4.40	1.29	0	6.06	1.45	0
		Range	0-30	0-4	0-30	0-15	0	0-30	0-20	0	0-30	0-15	0
		Not drinking (N, %)	251 (15.0)	36 (54.0)	0	9 (4.0)	18 (78.0)	0	44 (7.0)	72 (80.0)	0	38 (20.0)	34 (72)
	Average quantity of alcohol (g) per day when drinking beer	M	28.36	33.73	35.44	23.12	0	39.74	22.64	-	41.77	24.32	-
		SD	19.46	24.75	19.59	11.31	0	25.01	13.64	-	27.53	14.65	-
		Range	0-153.6	0-96	12.67-96	12.67-57.6	0	0-153.6	0-115.2	-	12.7-115.2	12.7-96.0	-
		Not drinking (N, %)	776 (48.0)	49 (73.0)	12 (18.0)	82 (37.0)	22 (96.0)	58 (28.0)	286 (46.0)	90 (100)	30 (32.0)	100 (52.0)	47 (100)
	Average quantity of alcohol (g) per day when drinking wine	M	33.60	37.50	34.18	31.00	5.87	39.77	32.10	-	42.45	32.88	4.4
		SD	15.02	14.34	16.10	13.04	5.08	19.34	12.89	-	19.34	13.76	6.2
		Range	0-105.6	17.6-70.4	8.8-105.6	17.6-88.0	0-8.8	8.8-88.0	8.8-88.0	-	17.6-88.0	17.6-70.4	0-8.8
		Not drinking (N, %)	637 (39.0)	44 (66.0)	22 (34.0)	42 (19.0)	20 (87.0)	79 (38.0)	173 (28.0)	90 (100)	44 (46.0)	78 (41.0)	45 (96.0)
	Average quantity of alcohol (g) per day when drinking spirituous beverages	M	18.98	27.57	22.79	14.00	2.64	31.39	14.37	8.16	28.94	14.94	8.21
		SD	18.16	21.08	15.37	12.13	3.73	28.09	10.93	2.76	20.52	10.58	2.78
		Range	0-179.52	5.28-63.36	5.28-84.48	0-73.92	0-5.28	5.28-179.52	0-73.92	5.28-10.56	0-105.6	0-52.8	5.28-10.56
		Not drinking (N, %)	1045 (64.0)	58 (87.0)	24 (37.0)	131 (60.0)	21 (91.0)	99 (48.0)	401 (64.0)	79 (88.0)	49 (52.0)	145 (76.0)	38 (81.0)
	Average quantity of alcohol (g) per day when drinking mixed beverages	M	20.35	17.16	22.88	15.39	10.56	29.61	17.15	10.56	33.94	19.56	7.04
		SD	14.19	7.86	17.09	10.03	-	18.31	7.14	0	26.58	12.32	6.10
		Range	0-105.6	10.56-31.68	10.56-84.48	0-52.8	10.56-10.56	10.56-84.48	10.56-42.24	10.56-10.56	10.56-105.6	10.56-63.36	10.56-31.68
		Not drinking (N, %)	1370 (84.0)	59 (88.0)	47 (72.0)	185 (84.0)	22 (96.0)	162 (78.0)	522 (84.0)	83 (92.0)	81 (85.0)	165 (86.0)	44 (94.0)
	Average quantity of alcohol (g) per day when drinking alcohol	M	45.38	27.69	72.22	47.69	1.45	76.32	43.32	1.82	68.93	37.59	2.21
		SD	37.38	40.01	38.77	28.19	3.34	44.00	28.35	3.81	42.57	31.52	4.01
		Range	0-251.52	0-148.8	17.6-226.56	0-186.24	0-10.56	12.67-251.52	0-201.92	0-10.56	12.67-187.97	0-187.97	0-10.56
		Not drinking (N, %)	252 (15.46)	37 (55.22)	0	9 (4.09)	19 (82.61)	0	44 (7.06)	72 (80.00)	0	36 (18.75)	35 (74.47)

Claudia Helmert*[1], Toni Fleischer[1], Sven Speerforck[1], Christine Ulke[1], Laura Altweck[2], Stefanie Hahm[2], Holger Mühlan[2], Silke Schmidt[2], Hans J. Grabe[3], Henry Völzke[4], Georg Schomerus[1]
[1]Department of Psychiatry and Psychotherapy, University of Leipzig Medical Center, Leipzig, Germany
[2]Department of Health and Prevention, University of Greifswald, Greifswald, Germany
[3]Department of Psychiatry and Psychotherapy, Greifswald University, Medical Center, Greifswald, Germany
[4]Institute for Community Medicine, Greifswald University, Medical Center, Greifswald, Germany
*Corresponding Author: Claudia Helmert, claudia.helmert@medizin.uni-leipzig.de

					Lifestyles [a]								
	Descriptives		*Total*	*Missings*	1	2	3	4	5	6	7	8	9
		N	1630	67	65	220	23	208	623	90	95	192	47
Variables		%		4.11	3.99	13.50	1.41	12.76	38.22	5.52	5.83	11.78	2.88
Physical activity	Much more physically active than other people my age	N	26	2	0	7	0	1	11	3	0	2	0
		%	1.60	2.99	0	3.18	0	0.48	1.77	3.33	0	1.04	0
	More physically active than other people my age	N	419	10	1	86	7	7	215	28	4	48	13
		%	25.71	14.93	1.54	39.09	30.43	3.37	34.51	31.11	4.21	25.00	27.66
	As physically active as other people my age	N	850	30	47	97	16	136	286	59	61	84	34
		%	52.15	44.78	72.31	44.09	69.57	65.38	45.91	65.56	64.21	43.75	72.34
	Less physically active than other people my age	N	263	14	16	24	0	52	93	0	22	42	0
		%	16.13	20.90	24.52	10.91	0	25.00	14.93	0	23.16	21.88	0
	Much less physically active than other people my age	N	66	5	1	6	0	12	18	0	8	16	0
		%	4.05	7.46	1.54	2.73	0	5.77	2.89	0	8.42	8.33	0
	Missings	N	6	6	0	0	0	0	0	0	0	0	0
		%	0.37	8.96	0	0	0	0	0	0	0	0	0

[a] Lifestyles: 1 – high SES, unhealthy; 2 – high SES, moderately healthy; 3 – high SES, healthy; 4 – middle SES, unhealthy; 5 middle SES, moderately healthy; 6 - middle SES, healthy; 7 – low SES, unhealthy; 8 – low SES, moderately health; 9 – low SES, healthy

Claudia Helmert[*1], Toni Fleischer[1], Sven Speerforck[1], Christine Ulke[1], Laura Altweck[2], Stefanie Hahm[2], Holger Mühlan[2], Silke Schmidt[2], Hans J. Grabe[3], Henry Völzke[4], Georg Schomerus[1]
[1]Department of Psychiatry and Psychotherapy, University of Leipzig Medical Center, Leipzig, Germany
[2]Department of Health and Prevention, University of Greifswald, Greifswald, Germany
[3]Department of Psychiatry and Psychotherapy, Greifswald University, Medical Center, Greifswald, Germany
[4]Institute for Community Medicine, Greifswald University, Medical Center, Greifswald, Germany
[*]Corresponding Author: Claudia Helmert, claudia.helmert@medizin.uni-leipzig.de

Table S2.1: Hierarchical linear model comparison by sex of group mean standardized results with outcome help-seeking intentions (full-maximum-likelihood-estimation; index i: level 1-unit – survey participants, n=1630; index j: level-2-unit – lifestyles, m=9)

Model	I: Intercept- only (Null Model)						II: Random-Intercept						III: Random-intercept & random Slope					
Help seeking$_{ij}$ =	$\beta_0 + \upsilon_{0j} + \varepsilon_{ij}$						$\beta_0 + \beta_{1j} shame_{ij} + \upsilon_{0j} + \varepsilon_{ij}$						$\beta_0 + \beta_{1j} shame_{ij} + \upsilon_{0j} + \upsilon_{1j} shame_{ij} + \varepsilon_{ij}$					
	overall		Men		Women		overall		Men		Women		overall		Men		Women	
	Coeff	SE	Coeff	SE	Coeff	SE	Coeff	SE	Coeff	SE	Coeff	SE	Coeff	SE	Coeff	SE	Coeff	SE
Fixed Effects																		
Intercept β_0	≈0	0.025	-.130 ***	.043	.120 ***	.033	.0001	.025	-.115 **	.043	.110 ***	.032	.0001	.025	-.109*	.047	.110 ***	.032
β_{1j} *Shame*							-.208 ***	.025	-.225 ***	.037	-.178 ***	.033	-.206 ***	.028	-.244 ***	.045	-.178 ***	.033
Random Effects																		
	Est.	SE	Est.	SE	Est.	SE	Est.	SE	Est.	SE	Est.	SE	Est.	SE	Est.	SE	Est.	SE
υ^2_{0j}	≈0	≈0	.003	.006	≈0	≈0	≈0	≈0	.003	.006	≈0	≈0	≈0	-	.006	.008	≈0	-
υ^2_{1j}													.001	-	.004	.008	≈0	-
Variance residuals/ overall error term ε_{ij}	.994	0.036	1.051	.056	.913	.044	.952	0.034	.999	.053	.883	.043	.951	-	.993	.053	.883	-
cov													≈0	-	-.005	.006	≈0	-
Intraclass correlation	ICC	SE	ICC	SE	ICC	SE												
	≈0	0	.002	.006	≈0	0												
	Chi bar²	p	Chi bar²	p	Chi bar²	P	Chi bar²	p	Chi bar²	p	Chi bar²	p	Chi bar²	P	Chi²	p	Chi²	p
Likelihood ratio test (comparing with one-level ordinary linear regression)	0	1	.25	.308	0	1	0	1	.30	.292	0	1	.08	.994	1.33	.722	0	1
							Level1	Level2	Level1	Level2	Level1	Level2	Level1	Level2	Level1	Level2	Level1	Level2
R^2 [a]							.043	.033	.050	-.038	.033	.908	.043	.033	.050	-.038	.033	.908
Model fit																		
AIC	4424.062		2072.032		2326.775		4355.577		2037.884		2298.306		4351.496		2040.852		2294.306	
BIC	4440.119		2085.749		2340.993		4376.985		2056.173		2317.259		4362.2		2068.286		2303.783	

[a] Bryk/Raudenbush (1992) R^2 , [b] Deviance: Dev=-2ln(L) vgl. Eid, Gollwitzer & Schmitt (2017)
***p≤0.001; **p<0.01; *p<0.05

An Explorative Cross-Sectional Analysis of Mental-Health Shame and Help-Seeking-Intention in different Lifestyles
Social Psychiatry und Psychiatric Epidemiology
Claudia Helmert[*1], Toni Fleischer[1], Sven Speerforck[1], Christine Ulke[1], Laura Altweck[2], Stefanie Hahm[2], Holger Mühlan[2], Silke Schmidt[2], Hans J. Grabe[3], Henry Völzke[4], Georg Schomerus[1]
[1]Department of Psychiatry and Psychotherapy, University of Leipzig Medical Center, Leipzig, Germany
[2]Department of Health and Prevention, University of Greifswald, Greifswald, Germany
[3]Department of Psychiatry and Psychotherapy, Greifswald University, Medical Center, Greifswald, Germany
[4]Institute for Community Medicine, Greifswald University, Medical Center, Greifswald, Germany
[*]Corresponding Author: Claudia Helmert, claudia.helmert@medizin.uni-leipzig.de

Table S2.2: Hierarchical linear model comparison by age of group mean standardized results with outcome help-seeking intentions (full-maximum-likelihood-estimation; index i: level 1-unit – survey participants, n=1630; index j: level-2-unit – lifestyles, m=9)

Model	I: Intercept- only (Null Model)						II: Random-Intercept						III: Random-intercept & random Slope					
Help seeking$_{ij}$ =	$\beta_0 + \upsilon_{0j} + \varepsilon_{ij}$						$\beta_0 + \beta_{1j} shame_{ij} + \upsilon_{0j} + \varepsilon_{ij}$						$\beta_0 + \beta_{1j} shame_{ij} + \upsilon_{0j} + \upsilon_{1j} shame_{ij} + \varepsilon_{ij}$					
	overall		≤61years		>61years		overall		≤61years		>61years		overall		≤61years		>61years	
	Coeff	SE	Coeff	SE	Coeff	SE	Coeff	SE	Coeff	SE	Coeff	SE	Coeff	SE	Coeff	SE	Coeff	SE
Fixed Effects																		
Intercept β_0	≈0	0.025	.060	.033	-.064	.038	.0001	.025	.071*	.032	-.073	.038	.0001	.025	.070*	.032	-073	.038
β_{1j} *Shame*							-.208 ***	.025	-.232 ***	.031	-.184 ***	.040	-.206 ***	.028	-.238 ***	.059	-.184 ***	.040
Random Effects																		
	Est.	SE	Est.	SE	Est.	SE	Est.	SE	Est.	SE	Est.	SE	Est.	SE	Est.	SE	Est.	SE
υ^2_{0j}	≈0	≈0	≈0	≈0	≈0	≈0	≈0	≈0	≈0	≈0	≈0	≈0	≈0	-	≈0	≈0	≈0	-
υ^2_{1j}													.001	-	.017	.016	≈0	-
Variance residuals/ overall error term ε_{ij}	.994	0.036	.896	.045	1.091	.056	.952	0.034	.839	.042	1.061	.055	.951	-	.826	.041	≈0	-
cov													≈0	-	≈0	.005	1.061	-
Intraclass correlation	ICC	SE	ICC	SE	ICC	SE												
	≈0	0	≈0	≈0	≈0	≈0												
	Chi bar²	p	Chi bar²	p	Chi bar²	P	Chi bar²	p	Chi bar²	p	Chi bar²	p	Chi bar²	P	Chi²	p	Chi²	p
Likelihood ratio test (comparing with one-level ordinary linear regression)	0	1	0	1	0	1	0	1	0	1	0	1	.08	.994	3.84	.279	0	1
							Level1	Level2	Level1	Level2	Level1	Level2	Level1	Level2	Level1	Level2	Level1	Level2
R^2 [a]							.043	.033	.065	-38.44	.028	-46.64	.043	.033	.065	-52.51	.028	-46.64
Model fit																		
DIC[b]																		
AIC	4424.062		2202.335		2214.14		4355.577		2148.309		2195.046		4351.496		2148.470		2191.046	
BIC	4440.119		2216.407		2228.02		4376.985		2167.067		2213.553		4362.200		2176.607		2200.300	

[a] Bryk/Raudenbush (1992) R^2 , [b] Deviance: Dev=-2ln(L) vgl. Eid, Gollwitzer & Schmitt (2017)
***p≤0.001; **p<0.01; *p<0.05

Claudia Helmert*[1], Toni Fleischer[1], Sven Speerforck[1], Christine Ulke[1], Laura Altweck[2], Stefanie Hahm[2], Holger Mühlan[2], Silke Schmidt[2], Hans J. Grabe[3], Henry Völzke[4], Georg Schomerus[1]
[1]Department of Psychiatry and Psychotherapy, University of Leipzig Medical Center, Leipzig, Germany
[2]Department of Health and Prevention, University of Greifswald, Greifswald, Germany
[3]Department of Psychiatry and Psychotherapy, Greifswald University, Medical Center, Greifswald, Germany
[4]Institute for Community Medicine, Greifswald University, Medical Center, Greifswald, Germany
*Corresponding Author: Claudia Helmert, claudia.helmert@medizin.uni-leipzig.de

Table S2.3: Hierarchical linear model comparison by having a mental illness or not of group mean standardized results with outcome help-seeking intentions (full-maximum-likelihood-estimation; index i: level 1-unit – survey participants, n=1630; index j: level-2-unit – lifestyles, m=9)

Model		I: Intercept- only (Null Model)						II: Random-Intercept						III: Random-intercept & random Slope					
Help seeking$_{ij}$ =		$\beta_0 + u_{0j} + \varepsilon_{ij}$						$\beta_0 + \beta_{1j}shame_{ij} + u_{0j} + \varepsilon_{ij}$						$\beta_0 + \beta_{1j}shame_{ij} + u_{0j} + u_{1j}shame_{ij} + \varepsilon_{ij}$					
		overall		Mental illness		No mental illness		overall		Mental illness		No mental illness		overall		Mental illness		No mental illness	
		Coeff	*SE*	*Coeff*	*SE*	*Coeff*	*SE*	*Coeff*	*SE*	*Coeff*	*SE*	*Coeff*	*SE*	*Coeff*	*SE*	*Coeff*	*SE*	*Coeff*	*SE*
Fixed Effects																			
Intercept	β_0	≈0	.025	.010	.043	-.001	.031	.0001	.025	.022	.044	-.018	.030	.0001	.025	.010	0.50	-.018	.030
Shame	β_{1j}							-.208 ***	.025	-.091*	.410	-.281 ***	.031	-.206 ***	.028	-.117*	0.057	-.281 ***	.031
Random Effects																			
		Est.	SE	Est.	SE	Est.	SE	Est.	SE	Est.	SE	Est.	SE	Est.	SE	Est.	SE	Est.	SE
	u^2_{0j}	≈0	≈0	≈0	≈0	≈0	≈0	≈0	≈0	≈0	≈0	≈0	≈0	≈0	-	.004	.009	≈0	-
	u^2_{1j}													.001	-	.010	.016	≈0	-
Variance residuals/ overall error term	ε_{ij}	.994	0.036	.981	.062	.994	.043	.952	0.034	.971	.061	.922	.040	.951	-	.960	.061	.922	-
	cov													≈0	-	.006	.010	≈0	-
Intraclass correlation		ICC	SE	ICC	SE	ICC	SE												
		≈0	0	≈0	≈0	≈0	0												
		Chi bar²	p	Chi bar²	p	Chi bar²	P	Chi bar²	p	Chi bar²	P	Chi bar²	p	Chi bar²	P	Chi²	p	Chi²	p
Likelihood ratio test (comparing with one-level ordinary linear regression)		0	1	0	1	0	1	0	1	0	1	0	1	.08	.994	.68	.877	0	1
								Level1	Level2	Level1	Level2	Level1	Level2	Level1	Level2	Level1	Level2	Level1	Level2
R^2 [a]								.043	.033	.010	.999	.073	1.000	.043	.033	.027	≈0	.073	1.000
Model fit																			
DIC[b]																			
AIC		4424.062		1437.686		2967.745		4355.577		1434.815		2888.910		4351.496		1438.133		2884.91	
BIC		4440.119		1450.378		2982.603		4376.985		1451.737		2908.717		4362.200		1463.516		2894.81	

[a] Bryk/Raudenbush (1992) R^2 , [b] Deviance: Dev=-2ln(L) vgl. Eid, Gollwitzer & Schmitt (2017)
***p≤0.001; **p<0.01; *p<0.05
